# Probiotic Yeasts: A Developing Reality?

**DOI:** 10.3390/jof10070489

**Published:** 2024-07-16

**Authors:** Vivian Tullio

**Affiliations:** Department of Public Health and Pediatrics, University of Turin, via Santena 9; 10126 Turin, Italy; vivian.tullio@unito.it

**Keywords:** yeast probiotics, promising benefit, probiotic development, genetic engineering

## Abstract

Yeasts are gaining increasing attention for their potential health benefits as probiotics in recent years. Researchers are actively searching for new yeast strains with probiotic properties (i.e, *Debaryomyces hansenii*; *Kluyveromyces marxianus*; *Yarrowia lipolytica*; *Pichia hudriavzevii*; and *Torulaspora delbrueckii*) from various sources, including traditional fermented foods, the human gut, and the environment. This exploration is expanding the pool of potential probiotic yeasts beyond the well-studied *Saccharomyces boulardii*. Research suggests that specific yeast strains possess properties that could be beneficial for managing conditions like inflammatory bowel disease, irritable bowel syndrome, skin disorders, and allergies. Additionally, probiotic yeasts may compete with pathogenic bacteria for adhesion sites and nutrients, thereby inhibiting their growth and colonization. They might also produce antimicrobial compounds that directly eliminate harmful bacteria. To achieve these goals, the approach that uses probiotics for human health is changing. Next-generation yeast probiotics are emerging as a powerful new approach in the field of live biotherapeutics. By using genetic engineering, scientists are able to equip these tools with specialized capabilities. However, most research on these probiotic yeasts is still in its early stages, and more clinical trials are needed to confirm their efficacy and safety for various health conditions. This review could provide a brief overview of the situation in this field.

## 1. Introduction

Probiotics are live bacteria that are good for human and animal health, especially the digestive system, and can help restore the proper balance of microorganisms in the gut. Because they support gut health, these microbes are frequently referred to as “good” or “helpful” microorganisms. For a long time, the discussion around probiotics has centered on lactic acid bacteria (LAB) like *Lactobacillus* and *Bifidobacterium*. These bacteria have been extensively studied and documented for their role in promoting a healthy gut microbiome, offering benefits like improved digestion, immune function, and even mental health [1,2,3].

However, a new frontier is emerging in the world of probiotics—the rise of probiotic yeasts. Many studies have suggested that certain yeast strains hold significant potential to join the ranks of established probiotic powerhouses [4].

## 2. Beyond Bacteria: Unveiling the Potential of Yeasts

In the last few decades, researchers have begun to explore the potential of yeast strains which have shown particular promise in supporting gut health [2]. Even though LAB will continue to play a significant role, these interesting new opportunities seem to have a positive impact on the future of gut health. Yeasts have been gaining increasing attention as potential probiotic candidates in recent years. While bacteria have dominated the probiotic market for a long time, research suggests that specific yeast strains possess properties that could be beneficial for human health [4].

Probiotic yeasts were generating excitement because they have interesting characteristics from an application point of view. (1) *Unique Advantages*: some probiotic yeasts might offer advantages over LAB in terms of survival. Studies suggest that certain yeast strains may be more resilient to the hostile environment of the stomach, reaching the intestines in higher numbers where they can exert their probiotic effects [2]. (2) *Diverse Functionality*: early research indicates that probiotic yeasts might have a broader range of functionality compared to some LAB strains. For example, some yeasts may produce compounds that not only benefit their own growth but also stimulate the growth of other beneficial bacteria in the gut [2]. This synergistic effect could lead to a more efficient and diverse gut microbiome. (3) *Appropriated Applications*: the discovery of various probiotic yeast strains opens doors for the development of more targeted probiotic interventions. Different yeast strains might be effective for addressing specific gut health concerns, offering a more personalized approach to probiotic supplementation [5].

## 3. *Saccharomyces boulardii*: A Pioneering Probiotic Yeast

As is known, *Saccharomyces cerevisiae* var. *boulardii* (often simply referred to as *S. boulardii*) has received the most scientific attention and is traditionally recognized as the probiotic yeast par excellence [4,6,7]. *S. boulardii* has been studied more extensively than other probiotic yeast strains. Research suggests that this yeast generally appears to be safe for most healthy individuals, and it may be effective in addressing issues like antibiotic-associated diarrhea, traveler’s diarrhea, acute infectious diarrhea, and even inflammatory bowel disease [6,8]. This is crucial for establishing a probiotic strain as a viable option for widespread use. Recent advancements in genetic engineering techniques are opening up possibilities for developing next-generation probiotic yeasts with enhanced probiotic properties or targeted functionalities [9,10].

## 4. Probiotic Yeast Effects

Understanding how yeast strains exert their probiotic effects is critical for selecting the most beneficial ones and maximizing their potential health impact.

This includes investigating their interaction with gut microbiota, immune modulation capabilities, and potential for improving gut health [11,12].

### 4.1. Interactions with Gut Microbiota

Probiotic yeasts may compete with pathogenic bacteria for adhesion sites and nutrients, thereby inhibiting their growth and colonization. Additionally, they might produce antimicrobial compounds that directly eliminate harmful bacteria [9,13,14].

The specific types of compounds identified so far can be categorized into two main groups:

Killer toxins (or zymocins): these are proteinaceous compounds produced by certain probiotic yeast strains, particularly those belonging to *S. cerevisiae* and Debaryomyces strains. Killer toxins target specific receptors on other yeast and fungal cells, creating pores that lead to cell death [15].

Mycocins: these are another group of antimicrobial compounds produced by some probiotic yeasts. Mycocins are extracellular proteins with a broader target range, meaning they can inhibit β-glucan synthesis, affecting the growth of other yeasts and molds [16]. In addition, mycocins produced by *Wickerhamomyces anomalus (Ascomycota, Saccharomycetes, Phaffomycetaceae) exert antimicrobial activity against Gram-negative bacteria such Acinetobacter baumannii* [17] *and Klebsiella pneumoniae* [18] *and Gram-positive bacteria, such as Staphylococcus aureus* [19].

The exact mechanisms by which mycocins work are still being elucidated, but they are believed to disrupt the cell membranes of target organisms [20]. In addition, mycocins are studied in the development of vaccines and used as epidemiological markers [16].

It is crucial to remember that investigations are still being conducted to fully define and describe the range of antimicrobial substances that probiotic yeasts release. Additionally, the effectiveness of these compounds can vary depending on the specific yeast strain and the target organism.

Probiotic yeasts can interact synergistically with beneficial gut bacteria, promoting their growth and activity. Probiotic yeasts have the ability to work in concert with good gut bacteria to enhance their development and activity. Improved nutritional absorption may result from increased nutrient degradation. Additionally, this “teamwork” stimulates the synthesis of beneficial metabolites like short-chain fatty acids, which support gut health by nourishing the intestinal lining [14,20,21,22].

Moreover, probiotic yeasts may influence the composition and diversity of gut microbiota, potentially enriching beneficial populations and reducing the abundance of harmful ones [20]. This shift in the microbial balance can significantly improve gut health and immune function [21,22].

### 4.2. Immune Modulation Capabilities

Probiotic yeasts play a multifaceted role in regulating the immune system within the gut. Beyond their competition with harmful bacteria, they interact with immune cells like macrophages and dendritic cells residing in the gut lining [23,24,25]. This interaction stimulates the production of cytokines and other immune mediators, such as interleukin (IL)-10 and interferon (IFN)-γ, that enhance the body’s overall defense mechanisms against invading pathogens [23,26].

In addition, probiotic yeasts may modulate the activity of immune cells involved in chronic inflammatory processes, potentially alleviating symptoms associated with inflammatory bowel disease (IBD) and other inflammatory gut disorders [21]. Studies suggest that probiotic yeasts can downregulate the production of pro-inflammatory cytokines like IL-1β and tumor necrosis factor-α (TNF-α), while promoting the production of anti-inflammatory mediators like IL-10 [23,26].

Probiotic yeasts seem to be also involved in allergy prevention. Early life exposure to certain *S.cerevisiae* strains might play a role in preventing food allergies and asthma in mice by influencing the development of the immune system [27,28]. Studies in animal models suggest that probiotic yeasts can regulate the balance between Th1 and Th2 immune responses, potentially turning the immune system towards a less allergic state [29]. However, more human trials are needed to confirm this benefit.

### 4.3. Overall Impact on Gut Health

Probiotic yeasts extend their influence beyond immune modulation, impacting various aspects of gut health. An important function of probiotic yeasts is that they can enhance the gut barrier, a complex system that acts as a physical and functional layer that protects the body from harmful substances and pathogens [30]. This can improve gut health and reduce the risk of infections. Probiotic yeasts can strengthen this barrier in several ways, e.g., by stimulating the production of tight junction proteins, which are essential for maintaining a strong and impermeable gut lining [31]. This tighter barrier reduces the risk of harmful substances and pathogens leaking from the gut into the bloodstream. Probiotic yeasts might promote the production of mucus by goblet cells in the gut lining [32]. This mucus layer acts as a lubricating and protective barrier, further enhancing gut defense mechanisms. Studies suggest that probiotic yeasts might be beneficial in preventing or alleviating symptoms of various gastrointestinal disorders, including diarrhea, constipation, and inflammatory bowel disease. Probiotic yeasts, specifically strains of *S. boulardii*, have demonstrated potential in mitigating the duration and intensity of diarrhea associated with antibiotic use [33,34,35]. They may also be beneficial in managing traveler’s diarrhea [36] and might improve bowel movement frequency in individuals with constipation [37]. As was previously mentioned, probiotic yeasts have the ability to regulate immune responses, which may lessen the inflammation linked to inflammatory bowel disease (IBD) [38]. Nevertheless, further investigation is required to ascertain their efficacy in the management of IBD.

By breaking down complex carbohydrates (sugars) that our bodies might find difficult to digest on their own, like those found in grains and legumes, and by producing digestive enzymes like lactase and β-glucosidases, probiotic yeasts can improve nutrient absorption and contribute to efficient digestion [4,22,39,40]. Moreover, probiotic yeasts ferment dietary fibers in the gut, leading to the production of beneficial short-chain fatty acids (SCFAs) like butyrate, acetate, and propionate [41]. These SCFAs serve as an energy source for gut cells, promoting gut lining health and nutrient absorption [42].

By thoroughly investigating these areas of functional characterization, researchers can gain valuable insights into the specific mechanisms by which different yeast strains exert their probiotic effects. This knowledge is essential for selecting the most promising candidates with unique functionalities for further development and ensuring their targeted application for optimal gut health benefits.

## 5. Yeast “New” Strains as Probiotics: Myth or Reality?

While research into probiotic yeasts is ongoing, there are not any definitively established “new” probiotic yeast strains yet [4]. However, the exciting part is that researchers are looking beyond *S. boulardii*. They are investigating the potential of various yeast genera like *Debaryomyces*, *Kluyveromyces*, *Yarrowia*, *Pichia*, and *Torulaspora*, isolated from fermented foods, traditional beverages, human microbiota, and natural sources [4,43,44,45].

These “new” probiotic yeast strains might offer a wider range of functionalities compared to existing probiotic options. They could potentially target specific gut health concerns or work synergistically with bacteria to enhance overall gut health [4].

Isolated from a variety of fermented foods and natural sources, strains of *Debaryomyces* yeast have been shown to have potential benefits, including tolerance to bile and stomach acid, which enables them to enter the intestines, and the generation of compounds that support gut microbiota [4]. Though intriguing, *Debaryomyces* as a validated probiotic is still in its early stages because it has not been as well researched as more well-known probiotics like *S. boulardii*. *D. hansenii*, which is well-known for its biotechnological uses, has outstanding antifungal qualities that can effectively prevent mold growth and, as a result, lower the level of aflatoxin contamination in food. Because of its ability to improve food safety, *D. hansenii* is a crucial topic for research in food microbiology and safety. Mold inhibition is caused by *D. hansenii* competing with other molds for vital nutrients and space, which reduces the amount of resources available for mold growth [46,47]. In areas where food products are kept, this competition is essential because it keeps mold colonies from growing and spreading. *D. hansenii* produces various metabolites that possess antifungal properties. These include organic acids, such as acetic acid and lactic acid, which lower the pH of the environment, creating conditions unfavorable for mold growth. Additionally, the production of volatile organic compounds (VOCs) like ethanol can also inhibit mold development. These small molecules are one of the numerous biological regulators of antagonistic interactions, which are important and highly effective against pathogens. Research indicates that *D. hansenii* can enzymatically degrade mycotoxins, including aflatoxins [46,47]. Enzymes such as laccases and oxidases produced by *D. hansenii* play a role in breaking down these toxic compounds, thus reducing their concentration in food products. Aflatoxins, produced primarily by Aspergillus species, are potent carcinogens and pose significant health risks. The ability of *D. hansenii* to reduce aflatoxin levels is of paramount importance in enhancing food safety. Studies have demonstrated that the presence of *D. hansenii* can lead to a significant decrease in aflatoxin production by inhibiting the growth of aflatoxigenic molds [47]. The antifungal properties of *D. hansenii* make it a valuable tool in the fight against mold contamination and aflatoxin production in food. By competing for nutrients, producing antifungal metabolites, and enzymatically degrading mycotoxins, *D. hansenii* could enhance food safety and extend the shelf life of food products. Continued research and application of this yeast in food systems hold promise for safer and more sustainable food preservation methods.

To validate its particular health benefits in humans, more research is required [4]. So, what is real and what is myth? As of right now, *D. hansenii* is neither. Probiotics like *Debaryomyces* strains have potential, but further study is required to make that potential a reality. Furthermore, just like bacteria, different strains of *Debaryomyces* may have varying probiotic capabilities. Finding the most effective strains is therefore essential [4].

Ochangco et al. [48] investigated the potential probiotic benefits of various *D. hansenii* strains that were isolated from cheese and the fish gut. The results indicated that the various traits of each strain might result in a range of probiotic effects. Although none of the strains exhibited the same level of stress resistance as *S. boulardii* strains, one strain survived well in the hostile gastric environment. Compared to *S. boulardii*, two strains produced a stronger anti-inflammatory response in immune cells, but one strain distinguished itself as the most promising probiotic candidate because of its ability to adhere to gut cells, survive in hostile conditions, and produce anti-inflammatory effects [48].

*Kluyveromyces*, *Yarrowia*, and *Torulaspora* are all exciting possibilities in the realm of new probiotic yeasts, but like *Debaryomyces*, they are still in the early stages of exploration.

Strains of *Kluyveromyces*, in particular *K. marxianus*, have been isolated from fermented foods, including kefir grain, fermented traditional dairy products, sewage from sugar businesses, and natural environments, such as plants and sisal leaves, showing promise for probiotic applications [4,49]. Early research suggests that *K. marxianus* produces a broad range of distinct metabolites that may be useful to the food and biotechnology sectors and that this yeast might be tolerant to stomach acid and bile, allowing it to survive the digestive tract and reach the intestines [4,49].

Similar to *Debaryomyces*, *Kluyveromyces* strains have not been extensively studied in humans. However, early research suggested that *K. marxianus* has potential benefits for gut health and immune function [50]. Recently, Nag et al. [51] observed in vitro that this yeast improved insulin sensitivity and reduced fat storage in fat cells, suggesting benefits for type 2 diabetes and obesity. Furthermore, this yeast showed cytotoxicity against colon cancer cells, suggesting anti-tumoral activity. Hence, these authors suggested that *K. marxianus* could have therapeutic potential. However, more research is needed to confirm their specific health benefits and identify the most effective strains [4,50,51].

*Yarrowia* is a relatively new genus being explored for potential probiotic properties [52]. Research for probiotic applications is even more limited compared to *Kluyveromyces*. Extensive investigation is needed to understand their safety and efficacy in humans [4]. For the past 20 years, the yeast *Y. lipolytica* has been used in industry to produce docosahexaenoic acid and eicosapentaenoic acid while adhering to good manufacturing procedures [53]. It has drawn notice recently for innovative biotechnological uses, like as an animal feed addition with functional properties. The productive and immunological characteristics of the animals given *Y. lipolytica* were improved, and their microbioma, fatty acid content, and biochemical profiles were also enhanced [54]. Some strains might possess characteristics like adhesion to the gut lining and antimicrobial activity against harmful gut pathogens [52,55]. Research has shown that this yeast is probiotic and beneficial to fish, birds, mammals, crabs, and mollusks. It is also harmless [54,56,57,58].

The *Pichia* sp. genus has probiotic potential among non-*Saccharomyces* yeasts [59,60]. *Pichia* probiotics are mostly obtained from food fermentation and have been shown to be able to thrive in gastrointestinal tracts. In addition to its probiotic functions as an antioxidant, the species *P. kudriavzevii* lowers cholesterol, has biological effects on the binding capacities of heavy metals, and improves the nutritional value of food [55,61,62].

Early research suggests that *Torulaspora* strains, particularly the species *T. delbrueckii*, have potential as a probiotic showing benefits like improving gut barrier function and modulating the immune system [4]. Studies suggest that *T. delbrueckii* may promote the growth of beneficial bacteria and inhibit harmful ones, leading to better digestion, reduced inflammation, and a stronger immune system [63]. Even if very little is known about its specific effects on gut health in humans, some *Torulaspora* strains exhibit additional benefits, such as antibacterial and antifungal activity against *Candida albicans*, *Escherichia coli*, *S. aureus*, and *Salmonella enterica* [4,64].

### Properties and Limitations of Probiotic Yeast Strains

While *S. boulardii*, *D. hansenii*, *K. marxianus*, *Y. lipolytica*, *P. kudriavzevii*, and *T. delbrueckii* possess several properties that make them promising as probiotics, including antimicrobial activity, enzyme production, and stress resistance, there are limitations to their introduction. These include safety concerns, lack of comprehensive human studies, regulatory hurdles, and the need for strain-specific evaluations. Addressing these limitations through rigorous research and regulatory processes will be essential for their widespread adoption as probiotics. Genetic engineering tools, particularly CRISPR/Cas9, have opened new possibilities for enhancing the beneficial properties of yeast strains used as probiotics and biocontrol agents. By overcoming current limitations through targeted genetic modifications, these yeasts can be optimized for improved efficacy, safety, and commercial viability [65]. *S. cerevisiae* and *S. boulardii* have been genetically engineered to enhance their probiotic properties, improving their efficacy and expanding their functional capabilities. According to a study, *S. cerevisiae* that expressed the flo11 gene—which codes for a flocculin on the cell surface—exhibited improved adhesion to intestinal epithelial cells. It is possible to increase the colonization and persistence of yeasts by engineering them to express adhesion factors that help them adhere to the mucosal surfaces of the gastrointestinal tract [66]. Furthermore, scientists have genetically modified *S. boulardii* and shown that this yeast is capable of secreting human lysozyme, which is advantageous for gut health [67]. In order to produce mutant yeast cells that can express recombinant protein and increase their survival rate under gastrointestinal stress conditions like low pH, high bile salt concentrations, and anaerobic conditions, Hudson et al. [68] modified *S. boulardii*. In addition, in a recent study by Kim et al., a strain of *S. boulardii* was engineered to metabolize L-fucose, a component of mammalian mucin [69]. Since VOCs produced by yeasts are important and highly effective against pathogens, engineering probiotic yeasts to increase the amount of VOCS could increase their benefits by preventing the growth of pathogens [55]. Probiotic yeasts can be engineered to secrete cytokines that modulate the host immune response, potentially improving immune system function. *S. cerevisiae* engineered to express and secrete interleukin-10 (IL-10) demonstrated anti-inflammatory effects in a mouse model of colitis [70]. *S. cerevisiae* has been modified to express enzymes that degrade quorum sensing molecules, thereby inhibiting the communication and virulence of pathogenic bacteria like *Pseudomonas aeruginosa* [71].

The main probiotic characteristics and limitations of the major yeast species described in this review are listed in Table 1. Additionally provided are some examples of how probiotic yeast strains have been improved by means of genetic engineering.

*S. boulardii* has the following probiotic qualities: (a) antimicrobial activity due to the production of antimicrobial compounds that inhibit pathogenic bacteria. (b) The ability to modulate immunity, which enhances immunity by inducing the synthesis of immunoglobulins. (c) The restoration of gut health since it supports the gut microbiome and maintains health, which is especially useful in the treatment of diarrhea. However, due to the possibility of fungemia, this yeast may not be appropriate for immunocompromised people, which could limit its use in sensitive populations. Furthermore, even though it is widely accepted as safe, different people may react differently [Table 1].

*D. hansenii* produces organic acids and other antimicrobial compounds that inhibit pathogenic bacteria and molds, showing good antimicrobial activity. It is known to produce killer toxins that have stable activity against pathogenic yeasts at 37 °C. Yeast killer toxins with stable activity at human body temperature could have medical applications. For instance, the use of concentrated purified toxin preparations in therapies against pathogenic yeasts is quite conceivable. Killer toxin production allows for the use of these yeasts as biocontrol agents in food production [Table 1].

It showed tolerance to salinity; hence, it thrives in high-salt environments, making it suitable for fermented foods like cheese and cured meats. Moreover, *D. hansenii* produces enzymes such as lipases and proteases, which can aid in digestion. Regarding possible limitations, there are safety concerns, as long-term safety studies of *D. hansenii* in the human intestine are still limited. Furthermore, it is not yet widely recognized or approved as a probiotic by regulatory bodies [Table 1].

*K. marxianus*’s probiotic properties concern its lactose utilization, which is efficiently fermented, making it beneficial for lactose-intolerant individuals. Moreover, its enzymatic production, like β-galactosidase, is crucial for supporting digestion. This yeast also currently has limitations due to both the limited studies in humans, most of the research being conducted in vitro or using animal models, and the fact that it is not widely accepted or approved as a probiotic in many areas [Table 1].

*Y. lipolytica* is capable of metabolizing lipids, which could have benefits for lipid digestion and absorption, and exhibits strong resistance to environmental stress, enhancing survival through the GI tract. Its limitations regard the fact that it could be a potential pathogen because some strains were observed to be opportunistic pathogens. Moreover, it requires more comprehensive safety and efficacy studies for use in humans [Table 1].

*P. kudriavzevii*, like some others, also has high tolerance to acidic and bile environments and produces compounds that inhibit the growth of pathogens. However, among the limitations, it requires thorough evaluation for safety and approval by food and health regulatory authorities [Table 1].

*T. delbrueckii*, because of its acid and bile tolerance, has the potential to be a probiotic by surviving the acidic conditions of the stomach and the bile in the intestines. Furthermore, it generates bioactive substances that inhibit pathogenic bacteria. However, they are less studied compared to other probiotic yeasts, with limited clinical trials, and their benefits may vary significantly between different strains [Table 1].

While probiotic yeast strains offer significant benefits, it is crucial to consider potential disadvantages, such as allergenicity. In fact, *S. cerevisiae*, commonly known as baker’s or brewer’s yeast, can be allergenic. Allergies to *S. cerevisiae* can cause symptoms such as respiratory issues, skin reactions, and gastrointestinal discomfort [88,89,90]. On the contrary, there is limited information on the allergenicity of *D. hansenii*. However, as with other yeasts, individual sensitivities can vary, and allergic reactions are possible [91]. Allergic reactions to *K. marxianus* are not reported. However, since the literature reports a rare but possible allergic reaction to *K. lactis*, another species of this genus, individuals with yeast allergies should be cautious when consuming products containing this strain [92]. Information on the allergenicity of *P. kudriavzevii* is sparse. Further research is needed to fully understand its potential to cause allergic reactions [93]. Likewise, there is minimal documented evidence of allergenicity for *C. milleri*. As a component of sourdough starters, it is generally considered safe, but allergic reactions cannot be entirely ruled out [94]. While probiotic yeast strains can provide numerous benefits in fermentation and biotechnological applications, awareness of potential allergenic effects is essential. Individuals with known yeast allergies should exercise caution and consult healthcare professionals when considering probiotic yeast consumption.

Other yeast species possess biocontrol properties, such as antimicrobial activity, competitive exclusion, and the production of bioactive compounds, which make them effective against various plant pathogens. However, limitations such as potential pathogenicity, regulatory hurdles, and the need for more comprehensive studies on their safety and efficacy must be addressed to fully harness their potential in food safety and probiotic/biocontrol applications (Table 2).

## 6. Fermentation Process: Boosting Nutrition and Flavor

Globally, interest in probiotic yeasts has surged in recent years and this is a rapidly evolving field. These yeasts are frequently associated with fermented food production, a traditional practice found worldwide [49]. The fermentation process not only enhances the taste and aroma of foods but also increases their nutritional value. Studies suggest that these yeasts might contribute to this enrichment by increasing B vitamins that are essential for energy production and metabolism; enhancing mineral bioavailability, making minerals more readily absorbed by the body, and breaking down complex carbohydrates, aiding digestion [105,106,107]. Indonesia is one of main country in the world that utilizes probiotics isolated from fermented foods and animal digestive tracts. Research, mainly in Indonesia, has explored incorporating probiotic yeasts into functional foods for poultry, improving their gut health, nutrient absorption, and overall well-being [44]. However, research on probiotic yeasts specifically for human applications seems less prevalent when compared to poultry [44]. The potential does not stop there. Studies suggest these yeasts might also serve as therapeutic agents for humans and animals suffering from dysbiosis, an imbalance of gut microbiota [2,108].

## 7. Commercial Formulas with Yeast Probiotics

As it is known, probiotics are live microorganisms that offer health benefits when consumed. They work by promoting the growth of good bacteria in the gut and inhibiting the growth of harmful bacteria. The applications of yeasts in human foods and animal feeds as well as in agriculture and other sectors are increasing and market demand is providing motivation to continue or even increase research and development in this field [109]. Probiotics are widely used by healthy people and in clinical settings, but there can be side effects. With new strains and uses in vulnerable groups, clear instructions are needed for safe and effective use. An international group met to discuss potential risks, including those for vulnerable people, and the importance of high-quality probiotics for these groups. They also stressed the need for reporting side effects and using whole genome sequencing to check probiotic safety. This will help scientists and physicians determine how safe probiotics really are [110].

While *D. hansenii* has potential as a probiotic, it is not yet widely used in commercial formulas, especially for humans. *Torulaspora*, similar to *D. hansenii*, is not yet a common ingredient in commercially available formulas. This is mainly due to the following factors:

(a) *Limited research in humans*. Most research on *D. hansenii* as a probiotic has been conducted on animals. While promising, human trials are needed to confirm its effectiveness and safety [111,112];
(b) *Focus on established strains*. Commercially available probiotic formulas often include well-studied strains like *Lactobacillus* and *Bifidobacterium* with a longer track record of safety and efficacy in humans;
(c) *Formulation challenges*. Yeast probiotics like bacteria probiotics might require specific processing or formulation techniques to ensure viability and delivery of its potential benefits. Some factors should be considered during processing and storage, such as temperature, pH, and various environmental aspects. These factors can damage the cells and reduce their viability during processing and storage [4,113].

Things can be different in the future. Alternative scenarios should be taken into consideration. These include combination formulas, where yeasts may be combined with established probiotic strains for a wider range of benefits, and/or specialized formulas [113].

While *Torulaspora*, and especially *T. delbrueckii*, exhibits probiotic potential comparable to that of *D. hansenii*, it is not yet frequently found as an ingredient in commercially available formulations. Improved intestinal health is one possible advantage [85].

## 8. Synergy with Bacteria: A Powerful Duo

Also very interesting could be the study of the synergy between bacteria and yeasts. Combining probiotic yeasts with existing bacterial strains in supplements could create a synergistic effect, enhancing the overall health benefits [43]. For instance, certain yeasts might produce compounds that promote the growth of beneficial bacteria. This collaboration can offer several advantages [114,115].

1. *Enhanced Microbial Growth*. Certain probiotic yeasts, like *S. boulardii*, may produce specific compounds such as prebiotics. These prebiotics act as food for beneficial bacteria strains like *Bifidobacteria* and lactobacilli, stimulating their growth and colonization in the gut [4];
2. *Improved Barrier Function.* Numerous investigations demonstrated a correlation between *S. boulardii* and a decreased level of Firmicutes and Proteobacteria in the gut microbiota and a greater proportion of Bacteroidetes. Additionally, by increasing the synthesis of short-chain fatty acids and inducing proinflammatory immune responses, this yeast can reduce inflammation [30]. Furthermore, *S. boulardii* has been shown by Kunyeit et al. to dramatically decrease the adherence of the multidrug-resistant species *C. auris* to the abiotic surface, suggesting that this would be a useful strategy for managing this yeast [116];
3. *Immune Modulation*. The combined effects of probiotic yeasts and bacteria could have a positive impact on the immune system. Studies suggest that this synergy might help regulate the inflammatory response and potentially reduce the risk of allergies or inflammatory bowel disease [12,30,43];
  *D. hansenii* is compatible with lactic acid bacteria (e.g., *Lactobacillus* spp. and *Lactococcus* spp.), hence it could be a component of starter cultures for lactic acid products such as cheese and yogurt. *D. hansenii* can enhance flavor development and improve the texture of the final product. It is capable of thriving in low pH environments, making it suitable for acidic fermentation processes. Certain strains of *D. hansenii* exhibit thermostability, which enhances its applicability in various thermal processing conditions. *D. hansenii* demonstrates high resistance to various stress factors, including osmotic stress, oxidative stress, and high salinity. In addition, it exhibits a high tolerance to various chemical agents, including preservatives and antifungal compounds, which underscores its robustness in industrial applications [112,117,118,119]. Furthermore, when cocultured with *Bacillus clausii*, *D. hansenii* has the ability to suppress the growth of this bacteria [120]. But the inhibition is related to different strains because it occurs at the strain level [120];
  - *S. cerevisiae* is compatible with acetic acid bacteria (e.g., *Acetobacter* spp.). It is used in the production of kombucha, where *S. cerevisiae* ferments the sugars to produce ethanol, which is then converted to acetic acid by acetic acid bacteria, contributing to the final flavor profile [121,122,123];
  - *P. kudriavzevii* is compatible with propionic acid bacteria (e.g., *Propionibacterium freudenreichii*) and could be used in the fermentation of dairy products and biotechnology industries [124,125];
  - *C. milleri* is compatible with lactic acid bacteria (e.g., *Lactobacillus sanfranciscensis*). *C. milleri* and *L. sanfranciscensis* work together to create the characteristic flavor and texture of sourdough bread through fermentation and acid production [126].

## 9. The Future

“New” probiotic yeasts could be incorporated into fermented foods or probiotic supplements, offering interesting possibilities for expanding the range of health benefits we can achieve through our diet and gut health [30,43]. Traditionally, probiotics have been associated with fermented dairy products like yogurt. Probiotic yeasts could be introduced into a wider range of fermented foods, like kimchi, kombucha, or even sourdough bread [127]. This would create an increased variety of fermented foods and more options for people with lactose intolerance or those who simply prefer different flavors. Further possibilities are represented by developing specific fermentation processes using various probiotic yeast strains. This could lead to fermented foods designed to target certain health concerns, like gut inflammation or immune function [128,129,130]. Moreover, probiotic yeasts might offer advantages when it comes to delivering beneficial microbes to the gut. Some yeast strains may be more resilient to stomach acid and bile, allowing them to reach the intestines in higher numbers. Hence, a wider range of health benefits could be achieved. For example, (a) probiotic strains can improve gut health, helping to maintain a healthy balance of gut microbiota and potentially reducing digestive issues like diarrhea or constipation. (b) Some yeasts might stimulate the immune system, potentially reducing susceptibility to infections. (c) Probiotic yeasts could target specific health concerns, like allergies, skin conditions, or even mental health, developing more targeted benefits.

**Figure 1:**
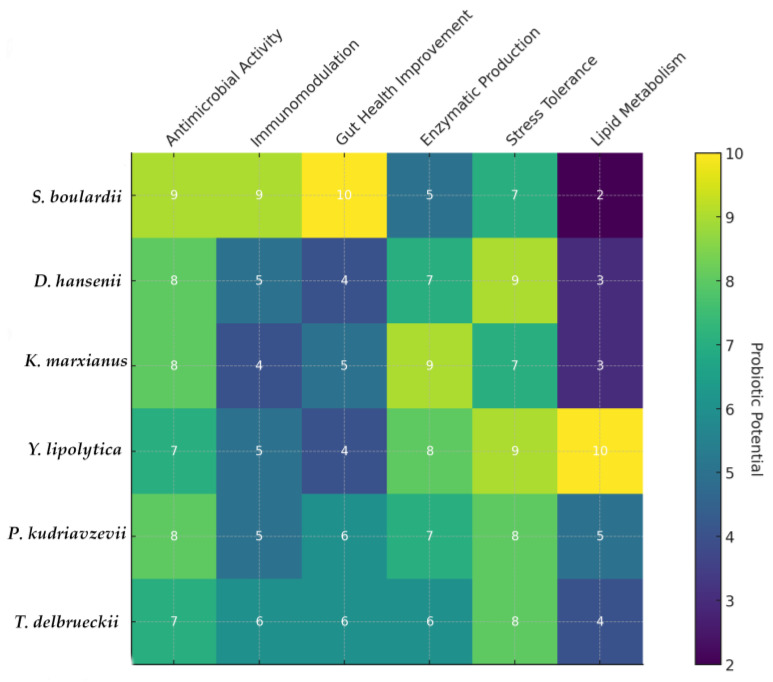
Probiotic potential of various yeast strains across different properties and the direction of development. Each cell represents the potential of a specific yeast strain in terms of antimicrobial activity, immunomodulation, gut health improvement, enzymatic production, stress tolerance, and lipid metabolism. The color intensity, from 2 to 10, indicates the level of probiotic potential, with higher values signifying greater potential.

To achieve these goals, the approach that uses probiotics for human health, to treat gut imbalances, is changing. Next-generation yeast probiotics are emerging as a powerful new approach in the field of live biotherapeutics [131,132]. These modified yeast strains go beyond the fundamental idea of probiotics. By using genetic engineering, scientists are able to equip these tools with specialized capabilities. Compared to conventional probiotics, this enables them to more effectively target particular health issues. For the time being, *S. boulardii* is one of the most promising next-generation yeast probiotics. Although this strain is currently utilized as a conventional probiotic to treat diarrhea, scientists are working to engineer it for additional uses [9,10,132]. Yeasts can be engineered to produce specific molecules that benefit the gut environment. For instance, they can be programmed to synthesize short-chain fatty acids that promote gut health, or modifications can be made to allow the yeasts to reach specific areas of the gut where they can exert their desired effects. The hostile environment of the gut can be challenging for probiotics; however, scientists are engineering yeast strains that are better able to pass through the digestive system and establish themselves in the gut. Probiotics derived from next-generation yeast may revolutionize the way gastrointestinal disorders are treated and perhaps even other medical conditions. Though the field is still in its early stages, ongoing research and clinical trials are paving the way for the development of more effective and targeted treatments (Table 1 and Table 2). The probiotic potential of various yeasts strains across different properties and the possible development direction are summarized in Figure 1.

*S. boulardii* excels in immunomodulation, gut health improvement, and antimicrobial activity. *D. hansenii* shows high potential in antimicrobial activity, stress tolerance, and enzymatic production. *K. marxianus* demonstrates strong enzymatic production and moderate potential across other properties. *Y. lipolytica* stands out in lipid metabolism and stress tolerance. *P. kudriavzevii* shows consistent potential across multiple properties, with notable stress tolerance and antimicrobial activity. *T. delbrueckii* is balanced across various properties but does not particularly excel in any single area.

This visual summary provides a clear overview of where each yeast strain excels and areas where further development could enhance their probiotic potential. (a) *Enhanced Functionalities*: focus on genetic engineering to enhance specific beneficial properties such as antimicrobial activity and stress tolerance. (b) *Safety and Efficacy*: comprehensive studies to ensure the safety and efficacy of these probiotic yeast strains for human consumption. (c) *Commercial Applications*: development of commercial probiotic products incorporating these optimized yeast strains. (d) *Regulatory Approvals*: navigating regulatory hurdles to achieve approval for new probiotic yeast strains in various regions.

## 10. Conclusions

Because the discovery of probiotic yeasts constitutes an important development in the probiotics field, the future of these probiotics is promising. Further research is needed to fully understand the specific health benefits these yeasts offer to humans and explore their applications in various food and therapeutic contexts. Several insights are necessary to move forward: (1) *Mechanism Elucidation*: understanding how probiotic yeasts interact with the gut microbiome and exert their health effects will pave the way for targeted applications. (2) *Strain Specificity*: not all strains within each genus will have the same probiotic potential. Identifying the most beneficial strains is essential. (3) *Clinical Trials*: studies in humans are needed to confirm the safety and effectiveness of yeast strains in promoting gut health.

This could lead to the development of novel probiotic supplements and functional foods promoting gut health and overall well-being for people around the world. However, the future of incorporating probiotic yeasts into fermented foods and supplements is definitely one to watch. It has the potential to revolutionize how we approach gut health and achieve a wider range of health benefits through diet and targeted interventions.

## Figures and Tables

**Table 1:** Main properties, limitations, genetic engineering tools, and examples of genetic engineering applications for each of the described yeast species.

Yeast Strain	Probiotic/Biocontrol Properties	Limitations	Genetic Engineering Tools and Genome Editing Technologies	Examples of Genetic Engineering Applications	Refs.
*Saccharomyces boulardii*	Antimicrobial activity Immunomodulation Gut health improvement (diarrhea treatment)	Not suitable for immunocompromised individuals (risk of fungemia) Variable individual responses	CRISPR/Cas9, TALENs, ZFNs, Homologous recombination	Engineering for enhanced stress tolerance, adhesion to intestinal cells and metabolic efficiency	[35,36,72]
*Debaryomyces hansenii*	Antimicrobial activity (organic acids, VOCs, killer toxins). Tolerance to salinity Enzymatic activity (lipases, proteases)	Limited safety studies Regulatory approval not widely obtained	CRISPR/Cas9, Homologous recombination, Plasmid-based expression	Engineering for enhanced lipase production for industrial applications	[15,16,45,46,47]
*Kluyveromyces marxianus*	Lactose utilization (beneficial for lactose intolerance) Enzymatic production (β-galactosidase)	Limited human studies Regulatory hurdles	CRISPR/Cas9, Homologous recombination, Plasmid-based expression	Engineering for improved lactose utilization and enhanced production of bioethanol	[73,74]
*Yarrowia lipolytica*	Lipid metabolism Stress resistance	Potential opportunistic pathogen Requires comprehensive safety and efficacy studies	CRISPR/Cas9, TALENs, Homologous recombination, Plasmid-based expression	Engineering for the production of higher levels of essential vitamins and for enhanced lipid accumulation for biofuel production	[75,76,77,78]
*Pichia kudriavzevii*	Stress resistance (acid and bile tolerance) Antimicrobial compounds	Pathogenicity concerns Regulatory and safety concerns	CRISPR/Cas9, Homologous recombination	Engineering for improved tolerance to environmental stresses and production of valuable metabolites	[79,80,81,82,83]
*Torulaspora delbrueckii*	Acid and bile tolerance Pathogen inhibition (bioactive compounds)	Limited research and clinical trials Strain-specific effects	CRISPR/Cas9, Homologous recombination	Engineering for improved fermentation efficiency and pathogen resistance	[63,84,85,86,87]

**Table 2:** Main properties, limitations, genetic engineering tools, and examples of genetic engineering applications for unconventional yeasts for their potential to serve as probiotics or biocontrol agents.

Yeast Strain	Probiotic/Biocontrol Properties	Limitations	Genetic Engineering Tools and Genome Editing Technologies	Examples of Genetic Engineering Applications	Refs.
*Candida oleophila*	Produces antifungal compounds Effective against post-harvest pathogens like *Penicillium* and *Botrytis*	Potential pathogenicity in humans Requires stringent regulatory approval	CRISPR/Cas9, Homologous recombination	Engineering for enhanced antifungal compound production and stress tolerance	[95,96,97]
*Hanseniaspora uvarum*	Inhibits a wide range of plant pathogens Produces volatile organic compounds	Can ferment at low temperatures Limited data on safety for human consumption	CRISPR/Cas9, Homologous recombination	Engineering for improved pathogen inhibition and enhanced fermentation characteristics	[98,99]
*Metschnikowia pulcherrima*	Produces pulcherrimin an iron-binding pigment Effective against various plant pathogens	Limited understanding of long-term effects Needs more studies on application in food systems	CRISPR/Cas9, Homologous recombination	Engineering for increased pulcherrimin production and improved biocontrol efficacy	[100]
*Rhodotorula glutinis*	Produces carotenoids and enzymes with antimicrobial properties Effective in biocontrol of post-harvest diseases	Opportunistic pathogen in immunocompromised individuals Regulatory and safety concerns	CRISPR/Cas9, Homologous recombination, Plasmid-based expression	Engineering for enhanced carotenoid production and stress tolerance	[101,102]
*Pichia anomala*	Broad-spectrum antifungal activity Effective in controlling spoilage molds and mycotoxin producers	Potential to produce harmful by-products Limited commercial application data	CRISPR/Cas9, Homologous recombination	Engineering for improved antifungal activity and reduced production of harmful by-products. Modifying strains for safer and more efficient use in commercial biocontrol products	[103,104]

## Data Availability

Not applicable.
